# Validity of a food frequency questionnaire for the estimation of total polyphenol intake estimates and its major food sources in the Japanese population: the JPHC FFQ Validation Study

**DOI:** 10.1017/jns.2021.25

**Published:** 2021-05-11

**Authors:** Nagisa Mori, Norie Sawada, Junko Ishihara, Ayaka Kotemori, Ribeka Takachi, Utako Murai, Masuko Kobori, Shoichiro Tsugane

**Affiliations:** 1 Epidemiology and Prevention Group, Center for Public Health Sciences, National Cancer Center, Tokyo, Japan; 2 Nutrition and Metabolism Branch, International Agency for Research on Cancer, Lyon, France; 3 School of Life and Environmental Science, Department of Food and Life Science, Azabu University, Kanagawa, Japan; 4 Department of Food Science and Nutrition, Faculty of Human Life and Environment, Nara Women's University, Nara, Japan; 5 Food Research Institute, National Agriculture and Food Research Organization, Ibaraki, Japan

**Keywords:** Dietary assessment, Food frequency questionnaire, Polyphenols, Validity

## Abstract

We examine the validity and reproducibility of a food frequency questionnaire (FFQ) in a subsample of participants in the Japan Public Health Center-based Prospective Cohort Study using a database of polyphenol-containing foods commonly consumed in the Japanese population. Participants of the validation study were recruited from two different cohorts. In Cohort I, 215 participants completed a 28-d dietary record (DR) and the FFQ, and in Cohort II, 350 participants completed DRs and the FFQ. The total polyphenol intake estimated from the 28-d DR and FFQ were log-transformed and adjusted for energy intake by the residual method. Spearman correlation coefficients (CCs) between estimates from the FFQ and 28-d DR as well as two FFQs administered at a 1-year interval were computed. Median intakes of dietary polyphenols calculated from the DRs were 1172 mg/d for men and 1024 mg/d for women in Cohort I, and 1061 mg/d for men and 942 mg/d for women in Cohort II. The de-attenuated CCs for polyphenol intake between the DR and FFQ were 0⋅47 for men and 0⋅37 for women in Cohort I and 0⋅44 for men and 0⋅50 for women in Cohort II. Non-alcoholic beverages were the main contributor to total polyphenol intake in both men and women, accounting for 50 % of total polyphenol intake regardless of cohort and gender, followed by alcoholic beverages and seasoning and spices in men, and seasoning and spices, fruits and other vegetables in women. The present study showed that this FFQ had moderate validity and reproducibility and is suitable for use in future epidemiological studies.

## Introduction

Polyphenols are present in most plant-based foods, including tea, coffee, cereals, pulses, fruit and vegetables^(1)^. Epidemiological studies to date have demonstrated that dietary polyphenol intake is associated with a reduced risk of certain types of cancer^(2,3)^, cardiovascular diseases^(4,5)^ and dementia^(6)^. These studies, conducted worldwide, have used either the U.S. Department of Agriculture (USDA) Database for the Flavonoid Content of Selected Foods^(7)^, or the Phenol-Explorer database to calculate total polyphenol intake^(8)^. However, given that some polyphenol-rich food items (e.g. soya foods) are widely consumed across Japan, we speculated that the main contributors of polyphenol in the Japanese population may differ from those in other parts of the world. Accordingly, total polyphenol intake is ideally estimated using a country-specific polyphenol database.

Several descriptive studies on polyphenol intake and their major food sources have been published, mostly from Europe^(9–14)^ and the Americas^(15–17)^. The main contributors to polyphenol intake in the European Prospective Investigation into Cancer and Nutrition (EPIC) Study population were shown to be coffee, tea and fruits^(9)^. Other contributors included chocolate, red wine and beans. In Asian settings, a few studies have been conducted in Japanese populations using the original polyphenol database^(18,19)^, in which the largest sources were shown to be coffee, followed by green tea, black tea, chocolate, beer and soya sauce^(18)^.

In the present study, we aimed to examine the validity and reproducibility of a food frequency questionnaire (FFQ) among subsamples of participants in the Japan Public Health Center-based Prospective Cohort Study (JPHC Study) using a Japan-specific database of polyphenol-containing foods^(20,21)^ and estimated the major food groups and items contributing to total polyphenol intake.

## Materials and methods

### Study population

The JPHC Study was established in the 1990s, and at present, all participants continue to be monitored. Participants of the present validation study included subsamples of the JPHC Study: The JPHC FFQ validation study. Details of the study design have been described previously^(22,23)^. At study recruitment, participants were aged 40–59 years in Cohort I and 40–69 years in Cohort II. In brief, in Cohort I, 215 participants completed a 28-d (14 d for the Okinawa area) dietary record (DR). The FFQ for validation analysis (FFQ_V) was administered few months after the completion of the DRs. Among the 215 participants, 209 completed the FFQ a second time (FFQ_R) for reproducibility analysis. Similarly, in Cohort II, 350 participants completed the DR and the FFQ_V for validation analysis, of whom 289 completed the FFQ_R for reproducibility analysis.

The JPHC FFQ Validation Study did not receive ethical approval as the study was conducted before the ethical guidelines for epidemiologic research put in place, which is now mandated in Japan. However, informed consent was obtained from the participants at recruitment. Additionally, the data use of the JPHC FFQ Validation Study was approved by the Institutional Review Board of National Cancer Center Japan.

### Database of total polyphenols

National Agriculture and Food Research Organization (NARO) constructed an updated version of polyphenol content of 162 foods, which included the publicly available NARO database^(20)^ and FCT 2015^(21)^ (Supplementary Table S1 of Supplementary material). Polyphenol content of each food item was measured using the Folin–Ciocalteu method by Japan Food Research Laboratories. In this table, the polyphenol content of chocolate products was taken from the FCT 2015; and that of onions, tomatoes, carrot and spinach listed was calculated as the mean of each item available in the existing NARO database. To calculate total polyphenol intake derived from the 28-d DR, we assigned the polyphenol content for an additional 261 food items based on the list of weight changes for each preparation method^(21)^, the ingredient blending ratio from food manufacturers, or substituted the existing polyphenol content with either: (1) different parts of same species, (2) similar species or (3) species with a similar polyphenol content.

### Nutritional calculation for DR and FFQ

Participants recorded the name of each dish and portion of food items and beverages consumed using the weighing scale. The food diary was then closely checked by a registered dietitian who coded each food item with a unique food number. Polyphenol intake was calculated by multiplying the polyphenol contents of the foods consumed and summing these values to derive a daily intake.

The FFQ used in the JPHC 5-year follow-up survey consisted of 138 food and beverage items and nine frequency categories, ranging between almost never to seven or more times a day. It was designed to capture the usual intake of 138 food and beverage items during the previous year. Polyphenol intake was estimated by multiplying the amount of polyphenol found in each food and beverage item by the reported frequency and portion size.

### Statistical analysis

The mean intake of polyphenols derived from the 28-d DR and FFQ were log-transformed and adjusted for energy intake by the residual method according to sex^(24)^. Medians and 10th–90th percentiles for the 28-d DR and FFQ were calculated and tested for differences using Wilcoxon two-sample tests. Spearman correlation coefficients (CCs) between estimates from the FFQ and 28-d DR were computed for both crude and energy-adjusted values. We then corrected the observed CCs for the attenuating effect of random intra-individual error for energy and polyphenols^(25,26)^. De-attenuation was calculated using the following formula: de-attenuated CC = observed energy-adjusted CC × SQRT (1 + *λx*/*n*), where *λx* is the ratio of intra- to inter-individual variation of DRs, and *n* is the number of DRs for each participant (28 d). The reproducibility of intakes was also assessed between the two FFQs administered at a 1-year interval.

To calculate the cohort-specific contribution of each food group to overall polyphenol intake, the percentage of total polyphenol intake for individual food groups from the DR was calculated, and then, these food groups were summed to derive cumulative polyphenol intake. The same analysis was repeated for individual food items. All analyses were performed using SAS (version 9.3, SAS Institute Inc., Cary, NC, USA).

## Results

The basic characteristics of subjects have been described elsewhere^(22,23)^. Median and 10th–90th percentile intake of total polyphenols were assessed using the 28-d DR and FFQ_V, and their correlation is described in Table 1. Regardless of cohort and gender, the intake of total polyphenols was significantly higher when assessed by FFQ than by DR (*P* < 0⋅003). The de-attenuated CCs for polyphenol intake were 0⋅47 in men and 0⋅37 in women for Cohort I and 0⋅44 in men and 0⋅50 in women for Cohort II.

**Table 1.** Median (10th–90th percentile) intake of total polyphenol according to DR and FFQ_V and their correlations

	DR			FFQ_V				Spearman's correlation coefficient		
	Median	10th	90th	Median	10th	90th	*P*^a^	Crude	Energy-adjusted	De-attenuated^b^
Cohort I										
Total polyphenols (mg/d)										
Men (*n* 102)	1172	669	1925	1418	736	2835	0⋅002	0⋅57	0⋅47	0⋅47
Women (*n* 113)	1024	612	1599	1243	611	2591	0⋅003	0⋅37	0⋅37	0⋅37
Cohort II										
Total polyphenols (mg/d)										
Men (*n* 174)	1061	631	1670	1292	608	2087	0⋅0002	0⋅40	0⋅44	0⋅44
Women (*n* 176)	942	558	1377	1263	646	2877	<0⋅0001	0⋅44	0⋅49	0⋅50

FFQ_V, food frequency questionnaire for validation analysis; DR, dietary record.

a Calculated using Wilcoxon two-sample tests.

b De-attenuated CC = observed CC * SQRT(1 + *λx*/*n*), where *λx* is the ratio of within- to between-individual variance for a nutrient, and *n* is the number of weighed food records.

Table 2 shows the correlation between FFQ_V and FFQ_R administered at an interval of 1 year. No significant differences in polyphenol intake were found between the two FFQs in either cohorts or genders (*P* > 0⋅07). The energy-adjusted CC for polyphenol intake was 0⋅59 in men and 0⋅46 in women for Cohort I and 0⋅51 in men and 0⋅56 in women for Cohort II.

**Table 2.** Median (10th–90th percentile) intake of total polyphenol and their correlation between two FFQs, administered at an average interval of 1 year

	FFQ_V			FFQ_R				Spearman's correlation coefficient	
	Median	10th	90th	Median	10th	90th	*P*^a^	Crude	Energy-adjusted
Cohort I									
Total polyphenols (mg/d)									
Men (*n* 101)	1417	736	2835	1474	632	2823	0⋅94	0⋅65	0⋅59
Women (*n* 108)	1237	592	2659	1369	682	2303	0⋅24	0⋅60	0⋅46
Cohort II									
Total polyphenols (mg/d)									
Men (*n* 143)	1292	608	2087	1325	741	2720	0⋅07	0⋅57	0⋅51
Women (*n* 146)	1314	674	2749	1348	599	2711	0⋅68	0⋅59	0⋅56

FFQ_V, food frequency questionnaire for validation analysis; FFQ_R, FFQ for reproducibility analysis.

a Calculated using Wilcoxon two-sample tests.

As described in Table 3, the percentage of total polyphenol intake for individual food groups derived from the DR indicated that non-alcoholic beverages, seasoning and spices, and alcoholic beverages were the top three contributors in the male population of Cohort I, and non-alcoholic beverages, alcoholic beverages, and seasoning and spices, in Cohort II. In contrast, the respective contributors in women were non-alcoholic beverages, seasoning and spices, and other vegetables in Cohort I and non-alcoholic beverages, fruits, and seasoning and spices in Cohort II. The top five food groups accounted for about 70 % of estimated total polyphenol intake.

**Table 3.** Food groups contributing to total polyphenol intakes from the dietary records in two cohorts

Cohort I				Cohort II			
Food groups	Number of foods assigned	Polyphenol intake (%)	Cumulative polyphenol intake (%)	Food groups	Number of foods assigned	Polyphenol intake (%)	Cumulative polyphenol intake (%)
Men (*n* 102)				Men (*n* 174)			
Non-alcoholic beverages	9	44⋅5	44⋅5	Non-alcoholic beverages	10	52⋅7	52⋅7
Seasoning and spices	6	13⋅8	58⋅3	Alcoholic beverages	5	11⋅1	63⋅8
Alcoholic beverages	5	10⋅5	68⋅8	Seasoning and spices	7	8⋅7	72⋅5
Other vegetables	53	8⋅0	76⋅8	Other vegetables	53	7⋅2	79⋅6
Cereals	18	6⋅2	83⋅0	Fruits	44	5⋅5	85⋅2
Green leafy vegetables	37	5⋅1	88⋅2	Pulses	11	5⋅0	90⋅1
Pulses	10	4⋅8	93⋅0	Green leafy vegetables	40	4⋅0	94⋅2
Fruits	38	4⋅5	97⋅5	Cereals	22	3⋅7	97⋅9
Potatoes and starches	7	1⋅6	99⋅1	Potatoes and starches	7	0⋅7	98⋅6
Nuts and seeds	11	0⋅4	99⋅4	Algae	3	0⋅5	99⋅1
Algae	3	0⋅3	99⋅8	Confectioneries	4	0⋅5	99⋅5
Confectioneries	4	0⋅2	100⋅0	Nuts and seeds	14	0⋅5	100⋅0
Women (*n* 113)				Women (*n* 176)			
Non-alcoholic beverages	9	51⋅8	51⋅8	Non-alcoholic beverages	10	59⋅8	59⋅8
Seasoning and spices	6	13⋅5	65⋅3	Fruits	46	8⋅2	67⋅9
Other vegetables	52	8⋅3	73⋅6	Seasoning and spices	7	7⋅8	75⋅7
Fruits	40	6⋅8	80⋅4	Other vegetables	55	7⋅1	82⋅8
Green leafy vegetables	37	5⋅4	85⋅8	Pulses	11	5⋅6	88⋅4
Cereals	19	5⋅3	91⋅1	Green leafy vegetables	40	4⋅0	92⋅4
Pulses	10	4⋅8	96⋅0	Cereals	22	3⋅4	95⋅7
Potatoes and starches	7	1⋅7	97⋅6	Alcoholic beverages	5	1⋅7	97⋅4
Alcoholic beverages	5	0⋅8	98⋅5	Potatoes and starches	7	0⋅8	98⋅2
Confectioneries	4	0⋅7	99⋅1	Confectioneries	4	0⋅7	99⋅0
Nuts and seeds	11	0⋅5	99⋅6	Nuts and seeds	14	0⋅5	99⋅5
Algae	3	0⋅4	100⋅0	Algae	3	0⋅5	100⋅0

Table 4 shows the top fifteen individual food items contributing to total polyphenol intake based on the DRs. Green tea accounted for more than 30 % of total polyphenol intake regardless of cohort and gender. Second- and third-ranked contributors were beer and miso in the male population of Cohort I and beer and soya sauce in Cohort II; and in women, these were instant coffee and miso in Cohort I and instant coffee and soya sauce in Cohort II.

**Table 4.** Top fifteen food items contributing to total polyphenol intakes from the dietary records in two cohorts

Cohort I				Cohort II			
FCT food number	Food item	Polyphenol intake (%)	Cumulative polyphenol intake (%)	FCT food number	Food item	Polyphenol intake (%)	Cumulative polyphenol intake (%)
Men (*n* 102)				Men (*n* 174)			
16037	Green tea	30⋅3	30⋅3	16037	Green tea	35⋅3	35⋅3
16006	Beer	9⋅4	39⋅7	16006	Beer	9⋅5	44⋅7
17046	Miso (red)	6⋅9	46⋅6	17007	Soya sauce	6⋅1	50⋅8
17007	Soya sauce	6⋅7	53⋅2	16046	Instant coffee	6⋅1	56⋅9
1083	White rice (cooked)	4⋅1	57⋅3	16045	Coffee	5⋅8	62⋅7
16046	Instant coffee	4⋅0	61⋅3	16042	Oolong-tea	2⋅6	65⋅3
16042	Oolong-tea	3⋅7	65⋅0	4046	Natto	2⋅2	67⋅5
16045	Coffee	3⋅2	68⋅2	17046	Miso (red)	2⋅0	69⋅5
16047	Coffee-based drinks	2⋅8	71⋅0	16047	Coffee-based drinks	1⋅8	71⋅3
4046	Natto	2⋅3	73⋅3	6150	Bamboo shoots	1⋅5	72⋅8
7148	Apples	1⋅8	75⋅1	7148	Apples	1⋅4	74⋅3
6191	Egg plants	1⋅6	76⋅6	6191	Egg plants	1⋅4	75⋅6
6205	Bitter melon	1⋅2	77⋅9	1083	White rice (cooked)	1⋅3	76⋅9
4032	Tofu	1⋅2	79⋅0	6301	Japanese mugwort	1⋅2	78⋅1
16001	Japanese sake	1⋅0	80⋅0	4032	Tofu	1⋅2	79⋅3
	Others	20⋅0	100⋅0		Others	20⋅7	100⋅0
Women (*n* 113)				Women (*n* 176)			
16037	Green tea	31⋅9	31⋅9	16037	Green tea	40⋅9	40⋅9
16046	Instant coffee	7⋅1	38⋅9	16046	Instant coffee	7⋅6	48⋅4
17046	Miso (red)	6⋅6	45⋅5	17007	Soya sauce	5⋅5	53⋅9
17007	Soya sauce	6⋅6	52⋅1	16045	Coffee	5⋅2	59⋅1
16042	Oolong-tea	5⋅3	57⋅4	16042	Oolong-tea	3⋅0	62⋅0
16045	Coffee	4⋅5	61⋅9	4046	Natto	2⋅9	64⋅9
1083	White rice (cooked)	3⋅0	64⋅9	7148	Apples	2⋅0	66⋅9
7148	Apples	2⋅5	67⋅4	16044	Black tea	1⋅8	68⋅7
4046	Natto	2⋅5	69⋅9	17046	Miso (red)	1⋅8	70⋅5
16047	Coffee-based drinks	1⋅8	71⋅7	6150	Bamboo shoots	1⋅7	72⋅1
6191	Egg plants	1⋅6	73⋅3	16006	Beer	1⋅3	73⋅5
6084	Burdock	1⋅1	74⋅4	6191	Egg plants	1⋅2	74⋅6
4032	Tofu	1⋅1	75⋅5	4032	Tofu	1⋅1	75⋅8
6267	Spinach	1⋅1	76⋅6	7027	Satsuma	1⋅1	76⋅9
2017	Potatoes	1⋅0	77⋅6	1026	Bread	1⋅0	77⋅8
	Others	22⋅4	100⋅0		Others	22⋅2	100⋅0

FCT, food composition table.

## Discussion

In the present study, we examined the validity and reproducibility of dietary polyphenol intake estimated using an FFQ. Results showed the moderate correlations between DRs and the FFQ as well as between two FFQs. More than 30 % of total polyphenol was obtained from green tea in both genders, followed by beer in men and instant coffee in women.

Among previous studies, a Brazilian study conducted among pregnant women assessed the correlation between dietary polyphenol intake estimated from a newly developed 52-item FFQ and either 24-h recall or a DR as a reference method^(27)^. The correlation between the FFQ and 24-h recall compared with a 3-d DR after energy adjustment was 0⋅51 and 0⋅46 when, and reproducibility between the two FFQs showed the relatively high correlation of 0⋅73. In addition, a U.S. based study^(28)^ also reported high validity (*r* 0⋅63 in the de-attenuated models) between total polyphenol intake from 24-h recalls and FFQ, as did a Belgian study, which also reported a correlation of 0⋅70 when the 3-d food record and FFQ were compared^(29)^. Our results show good consistency with this previous report.

The median intake of total polyphenols (1172 mg/d in men and 1024 mg/d in women for Cohort I and 1061 mg/d in men and 942 mg/d in women for Cohort II) was relatively close to those previously reported for a European population within the EPIC study of 1177 mg/d in men and 1192 mg/d in women^(9)^. On the other hand, in the Americas, a Mexican population^(15)^ reported a slightly lower median polyphenol intake of 735 mg/d among elderly adults (≥60 years), and a Brazilian population^(16)^ who were also aged ≥60 years reported even lower intake of 367 mg/d in men and 345 mg/d in women. Moreover, together with the median intake, the main contributor of foods containing polyphenols was differed from the present study as most of the above studies utilised the Phenol-Explorer database to estimate polyphenol intake. This is purely because the list of foods containing polyphenols included in the Phenol-Explorer database was not identical to our country-specific database. The largest source of dietary polyphenol within that European population was coffee, except for the UK population, for which tea was the biggest contributor^(9)^. In the present study, in contrast, green tea was the primary source of dietary polyphenol followed by coffee, beer, miso and soya sauce. Green tea intake is exceptionally high in Asian countries. Green tea has the highest concentration of (−) epigallocatechin gallate, the most active polyphenol catechin among all kinds of tea^(30)^. Recent studies conducted in elderly Japanese men and women^(19)^ and in middle-aged women^(18)^ reported that the largest source of polyphenol was coffee, followed by green tea. The difference may be related to the characteristics of the study population, given that the present study was conducted in rural areas of Japan whereas Fukushima *et al.*^(18)^ recruited participants in the Tokyo metropolitan area and Taguchi *et al.*^(19)^ recruited retired employees of the coffee/beverage industry.

Strengths of the present study include the use of a country-specific polyphenol database that employed the same analytical method as that in the Standard Tables of Food Composition^(21)^ in Japan. Our database included food items that are widely consumed within the Japanese population (e.g. soya foods). The comparative study of flavonoid intake assessed with the USDA database^(7)^ and Phenol-Explorer database^(8)^ demonstrated that flavonoid intakes estimated according to various databases may substantially differ^(31)^. In addition, because polyphenol content may vary substantially, depending on numerous factors such as ripeness at the time of harvest, environmental factors, processing and storage^(1)^, the use of a local database was probably the most appropriate way to estimate dietary polyphenols in the Japanese population.

However, several limitations should also be noted. In the present study, we were unable to evaluate the validity or reproducibility of classes and subclasses of polyphenols due to the unavailability of data. Second, we were unable to consider the possible loss of polyphenols as a result of cooking and processing processes, although the Phenol-Explorer^(8)^, which has been used in many populations, accounts for the effect of cooking loss and processing. Third, given that some polyphenols are metabolised by intestinal microbiota^(32)^, further investigation using biomarkers such as human plasma or urine samples may be necessary for better understanding.

In the present study, the validity and reproducibility of the estimation of dietary polyphenol intake were reasonable. These results suggest that the future use of this FFQ in epidemiological studies is suitable. In the Japanese population, the largest contributor to total polyphenol intake was green tea in both genders, followed by beer in men and instant coffee in women.

## Supplementary material

For supplementary material accompanying this paper visit http://dx.doi.org/10.1017/jns.2021.25. click here to view supplementary material
