# Supplementary material for: Validity of a food frequency questionnaire for the estimation of total polyphenol intake estimates and its major food sources in the Japanese population: the JPHC FFQ Validation Study
Source: J Nutr Sci. 2021 May 11;10:e35. doi: 10.1017/jns.2021.25 (PMC8143878; doi:10.1017/jns.2021.25)
Supplement: Supplementary file 1 [file S2048679021000252sup001.pdf]

Table S1. Food composition table of total polyphenols used in the current study.

FCT food number	Food item	Polyohenol content (g/100g)
1026	Common wheat, bread, white	0.050
1039	Common wheat, "Udon" (thick wheat noodles), boiled	0.010
1048	Common wheat, yellow alkaline noodles, boiled	0.020
1063	Durum wheat, macaroni and spaghetti, dry pasta, uncooked	0.110
1083	Rice, paddy rice, non-glutinous, well-milled, raw	0.020
1085	Rice, short grain, paddy rice, brown, "meshi" (cooked rice)	0.030
1129	Buckwheat, dried noodles, uncooked	0.290
2003	Konjac, block, made from fine powder	0.005
2006	Sweet potato, tuberous root, without skin, raw	0.040
2010	Taro, "Satoimo", corm, raw	0.120
2017	Potatoes, tuber, raw	0.040
2023	Yam, Chinese yam, "Nagaimo", tuberous root, raw	0.030
4001	Adzuki beans, mature seeds, whole, raw	0.803
4032	Soybeans, tofu, "Momen-tofu" (regular tofu)	0.040
4040	Soybeans, tofu, "Abura-age" (fried thin slices of pressed tofu), uncooked	0.040
4046	Soybeans, natto, "Itohiki-natto" (fermented whole soybean)	0.420
5001	Almonds, raw	0.180
5005	Cashew nuts, oil-roasted and salted	0.197
5008	Ginkgo nuts, raw	0.060
5011	Chestnuts, Japanese chestnuts, boiled	0.100
5014	Walnuts, roasted	0.883
5017	Sesame seeds, whole, dried	0.157
5034	Peanuts, mature seeds, Virginia type, raw	0.673
6003	Chives (leaf, raw)	0.037
6007	Asparagus (young, raw)	0.078
6015	Edamame (raw)	0.053
6020	Peas (young, raw)	0.050
6022	Peas, snap peas, immature pods, raw	0.027
6026	Peas, green peas, canned in brine	0.017
6032	Okra, pods, raw	0.223
6038	Turnip, root, without skin, raw	0.000
6042	Turnip, pickles, salted pickles, root without skin	0.010
6048	Pumpkin and squash, winter squash*, fruit, raw ［*Syn. Pumpkin］	0.030
6052	Leaf mustard, "Karashina", leaves, raw	0.084
6054	Cauliflower, inflorescence, raw	0.026
6061	Cabbage, common, head, raw	0.000
6064	Cabbage, red cabbage, head raw	0.078
6065	Cucumber, fruit, raw	0.010
6066	Cucumber, fruit, pickles, salted pickles	0.000
6072	Leaf green, "Mizuna", leaves, raw	0.054
6084	Edible burdock, root, raw	0.342
6086	Spinach mustard, "Komatsuna", leaves, raw	0.028
6093	Sweet peppers, "Shishito", fruit, raw	0.203
6095	Perilla, "Shiso", leaves, raw	0.890
6099	Garland chrysanthemum, leaves, raw	0.192
6101	Water shield, young leaves, bottled in water	0.090
6102	Ginger, immature rhizome with stem, raw	0.088
6103	Ginger, mature rhizome, raw	0.180
6116	Zucchini, fruit, raw	0.020
6117	Water dropwort, stems and leaves, raw	0.143
6119	Celery, petiole, raw	0.010
6122	Japanese royal fern, fiddleheads, dried, raw	1.580
6124	Broad beans, immature beans, raw	0.093
6128	Japanese radishes, Daikon, sprouts, raw	0.100
6130	Japanese radishes, Daikon, leaves, raw	0.124
6134	Japanese radishes, Daikon, root without skin, raw	0.010
6136	Japanese radishes, Daikon, "Kiriboshi-daikon" (cut and dried Daikon root), raw	0.293
6150	Bamboo shoots, boiled	0.322
6153	Onions, bulb, raw [2]	0.047
6156	Onions, red onions, bulb, raw	0.072
6160	Green bok choy, leaves, raw	0.040
6172	Hot peppers, fruit, dried	1.030
6173	Chinese preserving melon, fruit, raw	0.000

6180	Corn, sweet corn, canned products, whole kernel style	0.037
6181	Corn, sweet corn, young ear, raw	0.054
6182	Tomatoes, fruit, raw [2]	0.022
6183	Tomatoes, cherry tomatoes, fruit, raw	0.033
6184	Tomatoes, canned products, whole, without salt	0.027
6185	Tomatoes, canned products, juice, with salt	0.030
6191	Eggplant*, Japanese type, fruit, raw ［*Syn. Aubergine］	0.144
6193	Eggplant*, western type, fruit, raw ［*Syn. Aubergine］	0.184
6207	Chinese chive, leaves, raw	0.040
6214	Carrot, regular (European type), root without skin, raw [2]	0.013
6220	Carrot, "Kintoki" (oriental type), root without skin, raw	0.018
6223	Garlic, bulb, raw	0.122
6226	Welsh onions, "Nebuka-negi" (large variety, blanching cultivation), leaves, raw	0.016
6228	Welsh onions, "Konegi" (small variety), leaves, raw	0.040
6230	Turnip green, "Nozawana", leaves, pickles, salted pickles	0.037
6233	Chinese cabbage, head, raw	0.011
6238	Basil, leaves, raw	0.547
6239	Parsley, leaves, raw	0.230
6245	Sweet peppers, fruit, green, raw	0.040
6256	Japanese butterbur, petiole, raw	0.208
6263	Broccoli, inflorescence, raw	0.050
6265	Sponge gourd, immature fruit, raw	0.026
6267	Spinach, leaves, all season, raw [2]	0.090
6272	Turnip green, "Mizukakena", leaves, raw	0.086
6276	Japanese hornwort, "Ne-mitsuba" (branched by covering with soil), leaves, raw	0.227
6280	Japanese ginger, "Myoga", spike, raw	0.024
6283	Brussels sprouts, head, raw	0.076
6286	Bean sprouts, alfalfa sprouts, raw	0.080
6287	Bean sprouts, soybean sprouts, raw	0.063
6289	Bean sprouts, black gram sprouts, raw	0.030
6291	Bean sprouts, mung bean sprouts, raw	0.037
6293	Nalta jute*, stems and leaves, raw ［*Syn. Tossa jute］	0.640
6296	Lily, bulb, raw	0.020
6301	Japanese wormwood, leaves, raw	2.607
6304	Peanuts, immature beans, boiled	0.407
6306	Japanese scallion, "Rakkyo", mature bulb, pickles, sweetened	0.013
6307	Japanese scallion, "Rakkyo", immature bulb, raw	0.020
6308	Leeks, bulb and leaves, raw	0.014
6312	Lettuce, head lettuce, crisp type, soil culture, head, raw	0.026
6313	Lettuce, head lettuce, butter type, leaves, raw	0.032
6314	Lettuce, green leaf lettuce, leaves, raw	0.084
6315	Lettuce, red leaf lettuce, leaves, raw	0.250
6317	East Indian lotus root, rhizome, raw	0.158
6320	Green onion, "Wakegi", leaves, raw	0.050
6322	Wasabi, rhizome, raw	0.044
6325	Bracken fern, fiddleheads, boiled	0.053
7006	Avocados, raw	0.068
7012	Strawberries, raw	0.168
7015	Figs, raw	0.026
7018	Citrus, "Iyo", juice sacs	0.062
7022	Mume*, "Umeboshi" (pickled and dried mume), salted pickles ［*Syn. Japanese apricots］	0.060
7029	Satsuma mandarins, juice sacs, normal ripening type, raw	0.060
7037	Olives, in brine, green	0.170
7040	Oranges, navel, juice sacs, raw	0.090
7049	Japanese persimmons*, nonastringent, raw ［*Syn. Kaki］	0.036
7054	Kiwifruit, green flesh type, raw	0.000
7062	Grapefruit, white flesh type, juice sacs, raw	0.070
7077	Watermelon, red flesh type, raw	0.000
7082	Plums, European plums, dried	0.630
7088	Pears, sand pears*, raw ［Syn. Nashi pears］	0.012
7097	Pineapple, raw	0.040
7105	Citrus, "Hassaku", juice sacs, raw	0.080
7107	Bananas, raw	0.052
7116	Grapes, raw	0.034
7134	Muskmelon, greenhouse culture, raw	0.020
7136	Peaches, raw	0.078

7143	Citrus, "Yuzu", juice, fresh	0.024
7148	Apples, without skin, raw	0.076
7156	Lemons, juice, fresh	0.020
9004	Algae, purple laver, dried, toasted	0.910
9005	Algae, purple laver, dried, seasoned and toasted	1.050
14001	Olive oil	0.030
14008	Rapeseed oil	0.002
15072	Bun with filling, "Korone" (horn-shaped bread), with chocolate cream filling [1]	0.000
15097	Biscuits, hard biscuits	0.070
15098	Biscuits, soft biscuits	0.050
15114	Chocolate, chocolate-covered biscuit [1]	0.400
15115	Chocolate, white chocolate [1]	0.000
15116	Chocolate, milk chocolate [1]	0.700
15131	Bun with filling, baked bun with chocolate cream filling, thin dough type [1]	0.100
15137	Chocolate, chocolate with almonds [1]	0.500
16001	Fermented alcoholic beverage, "Sake", regular	0.020
16006	Fermented alcoholic beverage, beer, pale	0.050
16010	Fermented alcoholic beverage, wine, white	0.030
16011	Fermented alcoholic beverage, wine, red	0.280
16036	Green tea, "Sencha" (common grade tea), tea	14.600
16037	Green tea, "Sencha" (common grade tea), infusion	0.160
16042	Fermented tea, Oolong tea, infusion	0.090
16043	Fermented tea, black tea, tea	16.300
16044	Fermented tea, black tea, infusion	0.107
16046	Coffee, instant coffee, granules	11.733
16048	Cocoa, pure powder [1]	4.100
16049	Cocoa, chocolate milk powder [1]	0.900
16055	"Mugi-cha" (roasted barley tea), infusion	0.000
17007	Soy sauce, "Koikuchi-shoyu" (common soy sauce)	0.380
17016	Vinegar, rice vinegar	0.030
17046	Miso, rice-koji miso, red type	0.400
17061	Spices, curry powder	1.320
17064	Spices, pepper, white, ground	0.320

1 Polyphenol content of chocolate products are also available in the the Standard Tables of Food Composition in Japan, Seventh Edition.

2 Mean of three food items were calculated based on the database available from the National Agriculture and Food Research Organization (NARO).

3 Other unspecified food items were additionally measured their polyphenol content in prior to this study.
